# Equid Assessment, Research and Scoping (EARS): The Development and Implementation of a New Equid Welfare Assessment and Monitoring Tool

**DOI:** 10.3390/ani10020297

**Published:** 2020-02-13

**Authors:** Zoe Raw, Joao B. Rodrigues, Karen Rickards, Joe Ryding, Stuart L. Norris, Andrew Judge, Laura M. Kubasiewicz, Tamlin L. Watson, Holly Little, Ben Hart, Rebekah Sullivan, Chris Garrett, Faith A. Burden

**Affiliations:** The Donkey Sanctuary, Sidmouth, Devon, EX10 0NU, UK; joao.rodrigues@thedonkeysanctuary.org.uk (J.B.R.); karen.rickards@thedonkeysanctuary.org.uk (K.R.); joe.ryding@thedonkeysanctuary.org.uk (J.R.); stuart.norris@thedonkeysanctuary.org.uk (S.L.N.); andrew.judge@thedonkeysanctuary.org.uk (A.J.); laura.kubasiewicz@thedonkeysanctuary.org.uk (L.M.K.); tamlin.watson@thedonkeysanctuary.org.uk (T.L.W.); holly.little@thedonkeysanctuary.org.uk (H.L.); ben.hart@thedonkeysanctuary.org.uk (B.H.); rebekah.sullivan@thedonkeysanctuary.org.uk (R.S.); chris.garrett@thedonkeysanctuary.org.uk (C.G.); faith.burden@thedonkeysanctuary.org.uk (F.A.B.)

**Keywords:** welfare assessment, equid, animal-based indicators, evidence-based practice, equid welfare, one health

## Abstract

**Simple Summary:**

Animal welfare is a global concern which receives close public scrutiny. Numerous non-profit and non-governmental organisations exist to address problems relating to poor animal welfare, but there are multiple challenges surrounding how animal welfare is measured and recorded. We focus on the specific challenges around assessing equid welfare worldwide, and identify how stakeholders in this field of work are often unable to collaborate or pool resources due to differences in the welfare assessment tools they use. There is a need for a single welfare assessment tool which can be used across multiple contexts, yet which can yield comparable datasets to coordinate the approach to, and understanding of, global equid welfare. In response, we developed the Equid Assessment, Research and Scoping (EARS) tool which builds upon previously validated techniques, but extends them in a new framework which is applicable to multiple contexts. We have developed nine protocols, based upon 19 welfare indicators, and we describe the process of development here. We present the results from field-trialling three of the most frequently used protocols during the course of our work, assessing equids globally in farms and working environments, and equids on sanctuaries and properties in the UK and Europe. We found that the tool offered an easy and relatively quick way of collecting welfare data across multiple contexts, and propose that if developed further, it could be adopted by other organisations working to assess, understand and improve equid welfare worldwide.

**Abstract:**

The assessment of animal welfare poses numerous challenges, yet an emerging approach is the consolidation of existing knowledge into new frameworks which can offer standardised approaches to welfare assessment across a variety of contexts. Multiple tools exist for measuring the welfare of equids, but such tools have typically been developed for specific contexts. There is no ‘one size fits all’ which means that resulting datasets are generally non-comparable, creating a barrier to knowledge-sharing and collaboration between the many organisations working to improve equid welfare around the globe. To address this, we developed the Equid Assessment, Research and Scoping (EARS) tool, which incorporates pre-existing validated welfare assessment methods alongside new welfare indicators to deliver a larger and more comprehensive series of welfare indicators than currently exists, creating a single resource that can be used to assess equid welfare in any context. We field-trialled three welfare assessment protocols within the EARS tool, and applied these to welfare assessment of equids in a variety of contexts across nineteen countries. The EARS tool proved a useful, versatile and rapid method for collecting welfare assessment data and we collected 7464 welfare assessments in a period of fifteen months. We evaluate the EARS tool and provide ideas for future development.

## 1. Introduction

Animal welfare science is a rapidly expanding field and public concern for animal welfare is increasing. The public and regulatory focus on welfare spans a range of settings within which animals are kept or used, including laboratory, farming and production systems, zoos and aquaria, companion animals, and working animals, in both domestic and international settings [1,2,3,4,5]. Animal welfare is a difficult concept to define [6,7], but it can be broadly recognised as an animal’s ability to experience complete mental and physical health, and be able to live without suffering in an environment provided or adapted by human beings [8,9]. It is recognised that animal welfare should be a central consideration in the management and use of animals for societal and economic reasons [10] and, consequently, a number of tools to understand and assess animal welfare have been developed [11,12,13].

There is currently no globally accepted method for assessing animal welfare, but animal-based measures are considered the most reliable indicators of an animal’s welfare state [14,15] and are commonly used [16,17,18]. Non-animal-based indicators (e.g., environmental, resource-based or behavioural measures) also provide important information about welfare [19,20,21,22,23,24]. The consolidation of existing knowledge into new frameworks can offer standardised approaches to welfare assessment in a variety of contexts [25]. This process yields comparable datasets, allowing cross-contextual analysis both within and between settings and between species. Access to larger datasets and analysis of global trends will allow the development of refined interventions to improve welfare, and develop scientists’ understanding of how to tackle welfare concerns at scale [26,27].

### Assessing and Understanding Welfare in Equids

Horses, donkeys and mules are found in multifarious contexts around the world, and the pressures for improving welfare across all sectors is increasing. While existing equid welfare assessment tools offer species- or context-specific solutions (see next section), assessments with a single set of welfare indicators will not be applicable in all circumstances, and depending on country or context, perceptions of assessors regarding what constitutes ‘good’ welfare will differ. We recognised the need for a standardised tool that incorporates a variety of welfare indicators which can be selected based on the environment and location of an equid. Several approaches have been developed for the assessment of equid welfare [see 28 for full review]; our aim was to build upon this work and develop a set of welfare assessment protocols for horses, donkeys and mules that could be directly applied to the multiple contexts across which we (and other animal welfare organisations) typically work. Further, to improve comparability of datasets between organisations working in the equid welfare sector, a standardised tool will greatly improve the ability to analyse and interpret welfare trends in a more joined-up approach and at a global scale. In this paper, we review the existing equid welfare assessment approaches and present information about how we developed a new welfare assessment data collection tool for the assessment of equid welfare, known as the Equid Assessment, Research and Scoping (EARS) tool. We describe the tool, how it was developed, and present results from initial field-trialling of the tool. We discuss the reliability and ease of use of the tool, consider its limitations and discuss how it could be developed further.

## 2. Background and General Summary

### 2.1. Review of Equid Welfare Assessment Tools

#### 2.1.1. Welfare Assessment in Equids: Species Assessed

A variety of tools exist for measuring the welfare of equids [28], the majority of which have been developed for the assessment of welfare in horses (*Equus caballus)* [29,30]. Other *Equidae* (donkeys *Equus asinus,* and mules *Equus asinus* × *Equus caballus* hybrids) are typically not the target species for existing equid welfare assessment tools. One exception is the AWIN protocol for donkeys [31], but this does not specifically target mules nor provide scope for the multifarious environments that donkeys and mules are found in around the world.

In the UK, a commonly used welfare assessment tool for horses is the AWIN Welfare Assessment Protocol for Horses [32], which aimed to develop a series of practical assessment protocols to quantify the welfare of several species of farmed or kept animals, including horses. This approach was based on the Welfare Quality project, which placed an emphasis on animal-based measures as an indicator for animal welfare [33], and only included a resource- or management-based measure when no suitable animal-based measure was available, or when it was deemed more sensitive and reliable than an animal-based counterpart [34]. The Welfare Quality project presented four animal welfare principles (Good feeding, Good housing, Good health and Appropriate behaviour) and within these principles highlighted twelve distinct, but complementary, animal welfare criteria. Using the four animal welfare principles, AWIN researchers aimed to develop a harmonised and scientific welfare assessment protocol for horses based on valid, reliable and feasible animal-based and resource-based indicators [32].

#### 2.1.2. Welfare Assessment in Equids: Context

Understanding the welfare status of equids in different contexts is challenging and there is no clear consensus regarding which contexts may prove the most challenging for equids in terms of their welfare. Here, we provide an overview of the main contexts for which equid welfare assessments have typically been developed, identify the tools currently available for each context, and highlight existing gaps.

(a) Working equids

There are an estimated 60 million horses, 46 million donkeys and 10 million mules working worldwide [35]. Few data exist to describe how many people rely on working equids globally, but Brooke (a UK-based NGO focusing on working equid welfare) estimates that “...working equine animals help approximately 600 million people globally...” [36]. In low- and middle-income counties (LMICs), working equids support the livelihoods of rural communities, contributing directly and indirectly to livelihoods, and benefit local communities as a whole [36,37]. The primary welfare assessment tool for assessing working equid welfare is the Standardised Equine-Based Welfare Assessment Tool (SEBWAT) [38]. SEBWAT provides a framework for collecting welfare data using 40 animal-based measures, four descriptive identifiers and free text capability [38]. SEBWAT evolved from the Working Equid Welfare assessment (WEWA), an approach developed by Brooke and the University of Bristol [39]. SEBWAT has been used extensively in the field by Brooke and their partners, and has been identified as a useful tool for the context of working equids in LMICs [38].

(b) Production equids

Equids are used for production around the world. Horses are typically farmed for meat [40,41], hormones [42,43] and a number of slaughter by-products such as gelatin, hair and keratin [44]. Donkeys are increasingly being used for milk production as a solution to feeding infants with cow’s milk protein allergies [45,46] and there is an increasing demand for donkey skin products [47,48], which has led to a rapidly growing interest and practice of establishing farms for donkey production, particularly in Asia [48]. In recent years, there has been increasing interest in the welfare of farmed animals; however, this has primarily focused on a few key species and overlooks other commercially important species such as sheep, goats, turkeys and donkeys. This was one of the main drivers for the European Animal Welfare Indicators (AWIN) project which resulted in the development of a specific welfare assessment protocol for farmed donkeys (60), which has been used to assess the welfare of donkeys in a variety of settings including rescue centres, dairy farms and therapy centres [49,50,51,52].

One limitation of the AWIN protocol is that it has been validated for donkeys over the age of one year, so is unable to provide information about foal welfare. Resource-based indicators such as housing and husbandry will also have a significant impact on the welfare of production animals, so appropriate indicators need to be included in any assessment. In most farming systems donkeys need to be housed in groups, but welfare can be compromised by incompatibility of companions, or competition for resources (e.g., food and space). Assessing the behaviour of donkeys within groups can therefore provide useful information about the animals’ welfare state.

(c) Sanctuary equids

Equids in sanctuary represent a unique population that have been somewhat overlooked in the objective assessment of welfare. Sanctuaries and rescue centres can vary widely in their capacity, objectives and management approach, but often have to deal with equids that have pre-existing health or behaviour issues, having removed or rescued them from situations where welfare has been compromised. There can be significant pressure on resources as demand for intakes to sanctuaries can exceed available capacity. At present, there is no welfare assessment tool specifically for equids in sanctuaries or rescue settings.

(d) Feral equids

The advent of motorised vehicles and increasing mechanisation of much of the developing world has led to an increasing population of ‘de-domesticated’ equids, including donkeys [53,54,55]. Some populations may have been living independently of humans for several decades, while in other places, free-roaming donkey populations may have only recently established [56,57]. In some areas, feral and free-roaming donkeys are able to live natural lives; they live in self-defined groups, are able to freely express their natural behaviour and adapt to local ecological conditions [58,59]. However, in other areas, they may be seen as ‘pests’, ‘invasive species’ or ‘vermin’ and are subject to aggressive measures for population control or eradication [53,60,61,62]. In both contexts, feral donkeys can suffer from significant welfare problems [59] but the assessment of welfare in these contexts is lacking. The approach to assessing and understanding the welfare of these equids is outside the scope of this review, but we highlight it here and suggest it is an important area for future research, especially as competition for land and human-wildlife conflict increases [63,64,65].

### 2.2. Comparability of Welfare Assessment Data

In summary, existing equid welfare assessment tools provide useful approaches for assessing welfare in either: (i) a specific context, (ii) a specific species, or (iii) a specific species in a specific context. There is increasing need for a robust and adaptable tool with the flexibility to measure the welfare of any equid, in any context, using a standardised approach that can yield comparable datasets. To address this need, we created the EARS tool, which is built upon previously validated techniques but extends them in a new framework which is applicable to equids in multiple contexts.

## 3. Materials and Methods

### 3.1. Ears Tool: Development

The EARS tool is a questionnaire-based method of collecting welfare assessment data, in a standardised and stratified way. Its primary purpose is to provide reliable information about the general health and welfare state of equids in any context worldwide. The EARS tool is designed to obtain individual information about an equid and its surrounding environment, or about a group of equids, through cumulative repetition. Its development was inspired by other existing welfare assessment tools, and used these as a starting-point for further development. We used existing questions from three already validated, established welfare assessment tools: AWIN [29,30,31,32], SEBWAT [38] and WEWA [66]. We took the existing questions and evolved them into questions that could be applied to assessing equid welfare in any context. This process of amalgamation and refinement resulted in a total of 290 questions, divided into 19 welfare indicators (see Table 1), which have previously been identified and recognised as having a substantial influence on welfare [29,32,33,67]. Each indicator is divided into categories containing a set of questions designed to gather the necessary information required to assess the equid’s welfare under that indicator.

The process of selecting the questions from the existing welfare assessment tools, and evolving them to suit multiple contexts, was led by an equine veterinarian (second named author) with decades’ experience in the health and welfare of equids globally. The process of question development was supported and reviewed by all authors, each of whom are specialists in equine welfare and behaviour, and include equine veterinarians, equine behaviourists, qualitative and quantitative research scientists, and animal traction experts. Question development and refinement was also assessed by a panel of expert welfare scientists, external to the author group. We ensured all questions and their subsequent development retained a scientifically validated approach by ensuring that they had (i) either been validated in already existing and established tools, and also that (ii) all questions were aligned to the ‘Five Domains’ model of animal welfare science [68]. The Five Domains model allows for a systematic assessment of external factors that contribute to an animal’s wellbeing, and which affect the internal or mental state of the animal.

Alongside question development, thought was given to the appropriate design of responses and how training would be given to ensure consistency between users of different EARS protocols, and between different data collectors. The majority of EARS questions offer a series of structured qualitative responses by presenting a predefined list of optional answers, akin to a multiple choice answer format. This allows data to be collected in a standardised way between different contexts, different protocols and between different users. The predefined answer options were developed through a collaborative process with the panel of experts, as described above. For those questions adapted from other tools, the panel experts revised the response options where necessary, increasing and/or adapting the number of answers available. Where necessary, space is provided for free-text: for example, a question asking the specific age of the equid will allow the user to enter the age, rather than choose from a pre-defined list of age categories. Furthermore, questions with a predefined list of response options typically also present the option ‘other,’ allowing the assessor to collect and note any other source of relevant information as free text. During the development and initial field-trialling of the EARS tool, any free text answers that were repeatedly given by different data collectors were added to the list of predefined option responses.

#### 3.1.1. Protocols

The base set of 290 questions can be organised into ‘protocols’ to suit the user, or research question. A protocol is a set of questions that relates to a particular context. For example, the “Working equids” protocol contains all the questions from the 19 welfare indicators (Table 1) that relate specifically to the welfare of working equids. Therefore, this protocol will include questions about working hours, rest periods and working equipment. As another example, the “Feral protocol” contains all questions that would be relevant to feral equids, for instance, how far is the nearest water point, are they in close proximity to human settlements, etc. Protocols can be created to suit the type of population under assessment, local conditions, the research or management questions or any other specific aims of the assessment.

All protocols follow a top-down approach to assessing welfare; that is, they start with questions about the general and progress to questions about specifics. For example, general questions might capture information about overall environmental conditions, housing etc., before progressing to more specific questions such as body condition score, or severity of wounds. All questions are interlinked, such that some will only appear if specific option(s) from previous linked question(s) are selected. For example, if a data collector selects “yes” to a foal being kept with its mother, a series of questions will then follow asking for more detail, including at what age the foal is weaned. As of December 2019, we have developed nine protocols (see Table 2).

#### 3.1.2. Guidelines

Each question is accompanied by a set of specific guidelines which describe in detail how to collect the necessary data for that question. Guidelines include a description and explanation of the question, and a detailed explanation of each of the options provided. This is to ensure that all data collectors are employing the same methods of assessment, scoring and to the same scale.

#### 3.1.3. Sampling

Rather than setting a prescribed number of assessments as a target, assessors are encouraged to collect as many assessments as feasible in the time they have available, and to select the equids to be assessed in a random manner (pseudorandom or haphazard is acceptable). We specifically do not recommend that a set sample size needs to be assessed, for the following reasons: (i) we do not use sample sizes figures proposed by AWIN [29] due to the risk of the population not being normally distributed; the populations we encounter may not be normally distributed and an assessor in the field would not be able to tell just by looking at the population; (ii) when we suggest target sample sizes for the number of assessments to complete, the assessor may believe that they will need to collect this number of samples for their data to have significance testing applied. This is counterintuitive in the field and leads to the assessor thinking that if they do not get all the equids no comparisons can be drawn, and there is a risk they may abandon the assessment process.

#### 3.1.4. Training

Observer assessment and rating of animal-based indicators to assess welfare is a standard approach, and has been found to be reliable [69]. Nonetheless, bias is always a risk with such an approach. We attempted to minimise this risk by developing a complete training programme for use of the EARS tool, to ensure minimum standards of competency for all users. The training course focuses on data collection and storage, use of software, health and safety; including biosecurity, as well as specific training processes throughout all the indicators. All users of the EARS tool must attend the training course and pass two training assessments (a written and a practical assessment) to demonstrate their proficiency in assessment, their ability to collect data correctly, knowledge of the guidelines, and ensure a minimum standard. Only trainees with a combined result higher than 80% will pass the assessment and be approved to collect data using the tool. Untrained assessors should not use the EARS tool as this will restrict the validity of the dataset. The number of training days required is determined by the protocol the assessor is using. The EARS training team are responsible for deciding if the assessor is ready to collect data for a specific protocol. Currently, EARS training is provided for the Donkey Sanctuary (TDS) employees and overseas project partners.

#### 3.1.5. Technology and Data Management

The major technology used for data collection using the EARS tool is Open Data Kit [70]. Data storage and end-user dashboards are supported through the Google Cloud Platform and Shiny from R-Studio [71,72] respectively. ODK is a recognised mobile data collection tool for resource-constrained environments that has been utilised in multiple organisations, including the World Health Organisation, the Jane Goodall Institute, USAID and many others. ODK is an Android-based application.

Forms for EARS data collection are written in XForms (an XML format for collecting data from web forms) and uploaded to a TDS ODK aggregate instance deployed on Google Cloud. Users (data collectors) then access EARS protocols (forms) through the ODK Collect app installed on an android device. Access to EARS protocol forms is controlled through unique usernames and passwords, which are allocated to users once they have completed training. Once forms are downloaded to a device, users work offline completing their data collection programme. This software provides extensive benefits to EARS users as it can operate in remote areas with restricted telecommunication connectivity.

**Figure 1:**
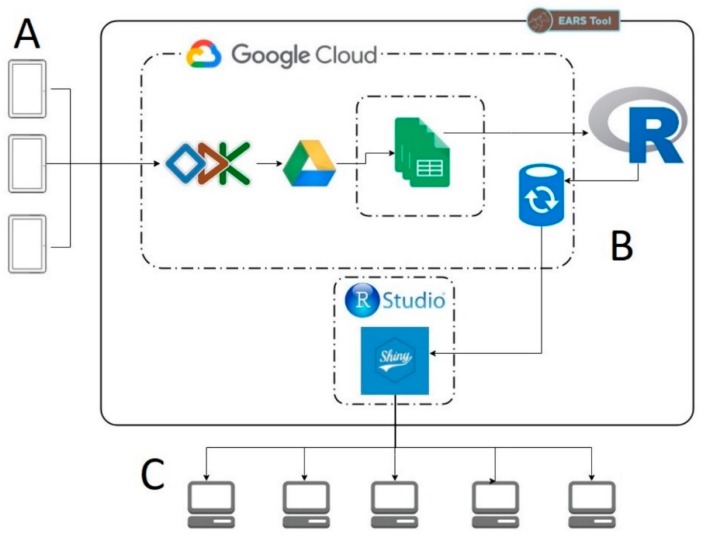
EARS data workflow with (**A**) representing data collection on an android device, (**B**) representing the cloud-based data storage and manipulation and (**C**) being the end-user interacting with a dashboard.

When users are connected to the internet, forms are submitted to ODK Aggregate. The ODK Aggregate form response data are then published in real time to Google Sheets within Google Drive. R programming language is used as part of an Extract, Transform and Load (ETL) process to read the Google Sheets and publish to a Shiny dashboard. As part of this process, the EARS information is also loaded into a PostgresSQL database. ETL processes are automated and performed daily through Windows Task Manager). The complete data collection and dissemination workflow can be seen in Figure 1. For users to utilise the EARS data workflow there are several hardware and software requirements that are outlined in Table 3.

EARS data are available for download from the EARS dashboard in comma-separated values (CSV) file format. Download is restricted to data relevant to the end-user (i.e., users only have access to data they collect). For global extraction and analysis, we use the PostgresSQL database. The EARS database includes personal data of users (data collectors), but not of owners nor people associated with the animals assessed. The only exception to this is the option to collect “owner ID” which allows data collectors to link an owner/user with a specific animal, for the purposes of associating an owner/user’s answers to social survey questions with the welfare of their equid. This “owner ID” is an anonymised identifier which does not contain any personal data. Through the creation and enforcement of roles and permissions (usernames, passwords and access limits) and the reduction of duplication, it is possible to make sure the EARS database is secure and compliant with general data protection regulation (GDPR) within the European Union (EU).

### 3.2. Ears Tool: Field Trailling

Following the initial development phase, we field-trialled the EARS tool to assess its efficacy and ease of use. As of December 2019, we developed nine protocols (see Table 2) to enable us to assess equid welfare in different contexts. Here, we focus on the three protocols that we have trialled most extensively: (1) TDS Farms, (2) Scoping, and (3) Production Farms.

All EARS assessments were carried out by either TDS staff (e.g., veterinary surgeons, Donkey Welfare Advisors, researchers) or global partners (i.e., Animal Nepal, Ayesha Chundrigar Foundation Pakistan, Greek Animal Welfare Fund). Everyone who carried out EARS assessments had received the same level of training, as detailed in the previous section. All assessors used the ODK app installed on an android device (usually a 10” Samsung tab A provided by TDS, but in some cases a TDS-owned or personal smartphone device) to collect data.

## 4. Results

### 4.1. Practicality of Use

We found that the EARS tool provided a suitable and practical approach for assessing and recording the welfare status of equids in the field for all three trialled protocols. Following successful completion of EARS training, all assessors were able to conduct welfare assessments to the required standard, were able to understand and use the EARS tool interface on the data collector devices, and the established data management processes worked as expected. Internet connectivity in the field was not an issue since assessors did not need to upload the data in real-time; rather, they worked offline and the data were submitted when the devices connected to Wi-Fi. ODK works offline, making the EARS tool independent of internet connectivity in the field; this solves one of the most limiting factors seen in other welfare tools.

### 4.2. Volume of Data

Using the three protocols, we collected a total of 7464 welfare assessments in nineteen countries (Table 4). We collected the highest number of assessments using the Scoping protocol, and the second highest using the TDS Farms protocol. We collected the fewest assessments using the Production Farms protocol (see Table 4). The full number of assessments collected are displayed by protocol and country in Table 5. The location and number of each assessment carried out at TDS locations are displayed in Table 6.

## 5. Discussion

This paper focuses on the development of the EARS tool as a method for collecting equid welfare assessment data. Consequently, we focus our discussion on the ease of use of the tool, and its ability to gather welfare assessment data across different locations and contexts, using the three protocols we have trialled most extensively to date. Detailed discussion of equid welfare status results is outside the scope of this manuscript, but will be analysed and presented in subsequent publications in the form of detailed, regional-specific case studies, and results discussed according to the context of each.

### 5.1. Field Trialling

The results from our field trials using three protocols across nineteen different countries (Table 4) demonstrates that the EARS tool can be applied to different contexts in which equids are found. We found that the tool could be used by both TDS staff and TDS partners following the completion of EARS training. It is unsurprising that the Scoping protocol yielded the greatest number of welfare assessments, since we had the greatest number of assessors trained for this protocol (*n* = 24; Table 4). It is also unsurprising that the TDS Farms protocols yielded the second-greatest number of welfare assessments, since our access to TDS owned and managed sites is unrestricted, and because welfare assessments using the TDS Farms protocol are conducted as standard during regular herd health assessments at all TDS sites. It was to be expected that the Production Farms protocol yielded the fewest assessments since numerous challenges exist in gaining access to production farms, especially if researchers are there to collect welfare data. That we were able to collect 277 assessments in three countries demonstrates how the Production Farms protocol can be successfully applied to this context, if access is provided by the farm owner. Despite the practical and logistical challenges of working in the field in LMICs, our TDS staff and TDS partners were able to complete 4175 assessments across ten countries (Table 4), indicating that the method is viable for use in such contexts.

### 5.2. Benefits of the EARS Tool

We found that when used by trained assessors, the EARS tool can be used to effectively collect welfare assessment data across different countries, contexts and management systems. Using portable Android devices made the data collection process easy, agile and highly adaptable, and the ODK data processing and management design took away the time requirement and risk of data transcription errors in data handling and storage. Providing a pre-defined set of responses to each question facilitated rapid assessment in the field during welfare assessments, improved user compliance, and reduced the likelihood of errors during the data entry process.

The EARS tool provides a robust and suitable method of assessing, baselining and monitoring equid welfare in a variety of contexts across the world. Development of such datasets can provide information and insight into which populations of equids might be suffering the poorest welfare, to inform decision-making processes about how charitable funds are spent, where to target interventions, or to strengthen evidence-based decision-making and practice. Being able to quantify welfare using a scientifically developed and recognised tool offers benefits to any user, over and above alternative methods or approaches.

### 5.3. Limitations of the EARS Tool

Comprehensive training is required to use the EARS tool, to ensure a minimum set of standards throughout the welfare assessment and data collection process. This mandatory period of training necessarily limits the speed at which new users can be deployed to use the EARS tool. However, once trained, EARS assessors can be rapidly deployed to any location. Future development of the EARS tool and the training process may include development of alternative ways of training users, including remote access, or online training.

At the end of each assessment, there is an “additional comments” section where the user can record any additional observations or contextual information. This section is free text, which limits the extent to which additional information can be included in any analysis. However, this is a common pitfall of free text and is not unique to the EARS tool.

### 5.4. The Importance of Asking the Right Question

A good research question defines the focus of any piece of research, and helps researchers determine which direction to take. In the field of welfare assessment, it is important to consider exactly what information you are looking for and exactly why knowing this information will support you in achieving your objectives. For example, if you are a decision-maker at an international animal welfare organisation (INGO) and you need to decide where the budget for the “improving equid welfare in Nepalese brick kilns” should be spent next year, you might want to know which populations of working equids in Nepal were suffering from the worst welfare, to decide where funds would be best spent to deliver sustainable impact. In which case, you would need a top-line overview of welfare status of each population your organisation worked with. However, if you are the manager of a UK-based equid sanctuary for example, you might want to understand the current status of all equids at all sanctuary sites, to understand how to prioritise resources, and to decide which interventions are most appropriate for each sanctuary site.

Ultimately, the EARS tool is a method for collecting data to answer a scientific question, or questions. It cannot provide definitive answers, but can generate data and evidence to consider alongside other factors. The ability of the EARS tool to answer questions around equid welfare will ultimately be influenced by the users’ formulation of a suitable question, and how the data will be used to inform policy, direct decision-making or influence outcomes. The EARS tool provides an efficient way to collect data, yielding raw data about equid welfare, but users should be able to analyse and interpret the data accordingly with respect to their research question or overall objective.

### 5.5. Future Development

The EARS tool was developed by TDS to provide a single, comprehensive welfare assessment approach which could be used across the multiple, global contexts in which we work. Although we have, to date, developed nine protocols, we have a bank of 290 questions divided into 19 indicators (Table 1), which enables us to build new protocols to suit new situations and contexts. The user interface and technology framework already exists, so future development of additional protocols is an easy process. There is scope to develop bespoke protocols to suit users’ needs, or to answer specific scientific research questions, and we anticipate developing a library of protocols which can be used in a standardised format, or be tailored as necessary.

## 6. Conclusions

Initially based on existing and validated welfare approaches [29,33,38], the EARS tool is now the most complete, complex and flexible set of questions available within one assessment tool, allowing the flexibility to assess the welfare of equids in almost any context globally, in a detailed, comprehensive and systematic way. We present this tool as a major step forward in the process of addressing a complex problem [8,13,73,74] in the context of assessing equid welfare.

## Figures and Tables

**Table 1:** List of the welfare indicators included in the Equid Assessment, Research and Scoping (EARS) tool.

	Indicator		Category
1	Initial information	a.	Sampling
		b.	Geographic data
		c.	Weather conditions
		d.	Assessor
		e.	Owner information
2	Housing	a.	Housing regime
		b.	Bedding
		c.	Water availability
		d.	Environment
		e.	Exercise regime
		f.	Additional information
3	Condition of assessment	a.	How the equid is observed at assessment
4	General identification	a.	Species being assessed
5	Specific identification	a.	Identifying information
6	Behaviour	a.	Animal’s general attitude
		b.	Owner or user/other animals
		c.	Interaction with/reaction to observer
		d.	Stereotypies
		e.	Foal behaviour
		f.	Fear and distress
		g.	Additional information
7	Specific identification	a.	Specific identification
		b.	Type of equid
		c.	Origin of equid
		d.	Identification system
		e.	Presence in the project
		f.	Food chain
		g.	Additional information
8	Working conditions	a.	Type of work
		b.	Equipment available
		c.	Vehicle
		d.	Harness
		e.	Pack/riding saddles
		f.	Bridle or similar
		g.	Working practices
		h.	Additional information
9	Harmful practices	a.	Attitudes/beliefs/traditions that negatively influence welfare
		b.	Additional information
10	End of life	a.	End of life
		b.	Additional information
11	Body condition	a.	Body condition score
		b.	Nutrition
		c.	Dentistry
		d.	Parasites
		e.	Additional information
12	Skin system	a.	Skin system alterations
		b.	Open wounds
		c.	Other skin lesions
		d.	Additional information
13	Musculoskeletal system	a.	Lameness
		b.	Hooves
		c.	Conformation and leg lesions
		d.	Vertebral column region
		e.	Additional information
14	Health status	a.	General health status
		b.	Mucous membrane
		c.	Body temperature
		d.	Pulse rate
		e.	Respiratory system
		f.	Ocular system
		g.	Faeces
		h.	Coat
		i.	Reproductive system
		j.	Prolapse
		k.	Abdominal pain
		l.	Foaling
		m.	Additional information
15	Additional location information	a.	General
		b.	Market
		c.	Slaughter
		d.	Dairy / Pharmaceuticals
		e.	Additional information
16	Transport	a.	Vehicle
		b.	Journey
		c.	Additional information
17	Habitat	a.	Environment
18	Feral Population	a.	Population
19	Final general questions	a.	Final general questions

**Table 2:** List of EARS tool protocols developed by the Donkey Sanctuary as of December 2019.

Protocol	Situational Application
TDS Farm Assessment	Equids on TDS owned farms
Scoping	Equids in any situation
Working equids	Equids in all working contexts
Harness	Any equid using a harness (carts and packsaddles)
Feral	Any feral population
DWA	Assessments of equids in UK (including TDS Guardian homes, markets, animal welfare investigations)
Production farm	Equids in production farms
Production farm (unweaned foal)	Unweaned foals in production farms

**Table 3:** Hardware and software requirements for the different data workflow stages within the EARS tool.

Workflow Stage	Hardware Required	Software Required	Comments
A: Data collection	Android phone or tablet	ODK Collect app, Android 4.0 or later	ODK Collect is installed through the Play Store
B: ETL processes	A personal computer	R and RStudio	This stage is not accessible to end-users
C: Accessing raw data	A personal computer, tablet or smartphone	Any modern web browser	The dashboards are web-based, with no specific hardware or software requirements beyond web access

**Table 4:** Summary of the welfare assessment data collected during field-trialling using each of the three protocols.

Protocol	No. of Assessments	First Date	Last Date	Number of Countries	Number of Different Assessors
Scoping	3898	08/08/2018	23/10/2019	7	24
DS Farms	3289	11/09/2018	05/11/2019	9	20
Production Farms	277	17/10/2018	25/07/2019	3	3

**Table 5:** Number of assessments collected using each of the three protocols, presented in descending order by country.

Protocol	Country	Number of Assessments Collected
DS Farms	United Kingdom	2906
	Ireland	194
	Cyprus	52
	Italy	38
	Spain	37
	Romania	34
	Greece	17
	Portugal	11
Scoping	Nepal	2648
	Pakistan	665
	Greece	335
	Peru	92
	Ghana	79
	India	64
	Burkina Faso	15
Production Farms	Italy	167
	People’s Republic of China	62
	Serbia	48

**Table 6:** Number of assessments collected using the TDS Farms protocol, listed by country and site.

Location	Number of Assessments Collected
**England**	
ENG-A	574
ENG-B	469
ENG-C	372
ENG-D	349
ENG-E	343
ENG-F	321
ENG-G	315
ENG-H	74
ENG-I	17
ENG-J	15
ENG-K	13
ENG-L	2
**Scotland**	
SCO-A	15
SCO-B	12
SCO-C	2
**Ireland**	
IRE-A	77
IRE-B	65
IRE-C	34
IRE-D	16
IRE-E	13
IRE-F	2
**Romania**	
ROM-A	32
ROM-B	2
**Cyprus**	
CYP-A	19
CYP-B	19
CYP-C	14
**Italy**	
ITA-A	20
ITA-B	18
**Greece**	
GRE-A	17
**Spain**	
SPA-A	20
SPA-B	17
**Portugal**	
POR-A	11
